# Closing the loop on female fertility

**DOI:** 10.1126/sciadv.abn1373

**Published:** 2021-12-15

**Authors:** Teresa K. Woodruff

**Affiliations:** Department of Obstetrics and Gynecology and Department of Biomedical Engineering, Michigan State University, East Lansing, MI, USA. Email: tkw@msu.edu

## Abstract

Discovery of a previously unidentified pituitary protein could provide innovative therapeutic options to regulate female fertility.

Human reproductive cycles depend on a careful choreography of pituitary and gonadal or sex hormones. These feedback loops drive hormonal health and the reproductive potential of individuals. By identifying the elusive inhibin B co-receptor, TGFBR3L, Brule *et al*. (*1*) provide a more complete understanding of hormonal and reproductive signaling pathways. Moreover, their findings herald new fertility diagnoses and interventions for both humans and animals.

In 1923, J. Motram and W. Cramer predicted the biology of a gonadally derived negative feedback loop when they observed that removal of mammalian testes resulted in hypertrophied or “castration” cells in the anterior pituitary (*2*). However, another 54 years would elapse before N. Schwartz and N. Channing, followed by other groups around the globe, redefined the anatomical observation when they showed that removal of the ovaries resulted in a rise of follicle-stimulating hormone (FSH) in females (*3*). This powerful physiological paradigm was strengthened by the simultaneous identification of the protein inhibin in the follicular fluid (*2*–*4*). Since 1977, reproductive research has pinpointed the signaling mechanisms underlying these early observations of the critical connections between pituitary and gonads (testes or ovaries), and now, Brule *et al*. (*1*) have identified the co-receptor for inhibin B. With this discovery, nearly 100 years in the making, the loop creating the most powerful negative feedback system responsible for reproductive success and disease has now been closed (Figure 1).

**Fig. 1.**
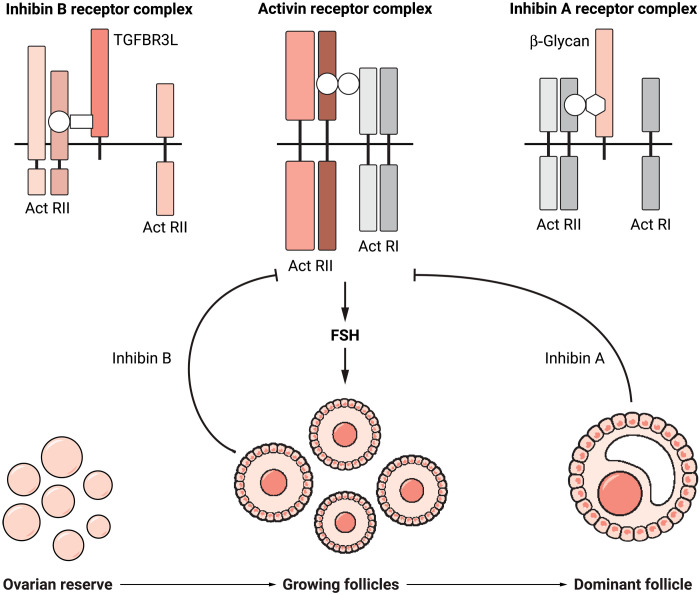
The molecular endocrinology and physiological outputs of the female reproductive cycle are coordinated by the pituitary gland, ovaries, and the hormones activin and inhibin. Activin is produced in the pituitary gonadotroph cell and stimulates FSH release. FSH, in turn, stimulates growth of maturing ovarian follicles from a quiescent ovarian reserve. FSH also activates inhibin B. The mass action of inhibin B by the large number of newly growing follicles inhibits activin and limits FSH production. Reduced FSH levels cause a winnowing of follicles to a single, dominant follicle that secretes inhibin A. Inhibin B and then inhibin A work through independent co-receptors to regulate FSH. Illustration credit: Ashley Mastin/*Science Advances*.

To better understand how this elusive, essential, and elegant system works, first, a bit of background on the reproductive endocrinology that defines the reproductive cycle and then a few thoughts on how the discovery of a specific receptor heralds new therapeutic targets for cases of infertility arising from unknown causes.

In a reproductively mature female, FSH from the anterior pituitary gland travels through the circulatory system to find its one and only target cell in the ovarian follicle. The follicle is the functional unit of the ovary, and women are born with a limited number (around 10,000). Small numbers of follicles grow in successive waves from the time of puberty until menopause, when follicles in the original reserve reach zero.

FSH is the hormone that does what its name says—it causes follicles to grow through a series of growth factors. The new follicles also have the responsibility of turning off FSH and do this by secreting the hormone inhibin B, which travels through the circulatory system to find its one and only target cell, the pituitary gonadotroph. Locking onto its target, inhibin B blocks an essential “on” switch, the paracrine hormone activin. When follicle numbers and therefore inhibin B are limited or lost at menopause, FSH levels rise and stay permanently elevated. In males, where sperm production is ceaseless and cycle-less, the testicular somatic cells produce inhibin B, which provides a constant negative feedback loop between gonad and pituitary. So simple and elegant! But why was the discovery of the inhibin co-receptor so arduous? It turns out that the female reproductive cycle requires a sequence of precisely timed hormones, and the tour de force of the Brule work is that the researchers used a variety of techniques to identify and demonstrate the unique way in which inhibin B works.

In each reproductive cycle, more ovarian follicles are activated than are naturally ovulated but are collectively necessary to produce sufficiently high and immediate inhibin B levels to rapidly and cycle-selectively block FSH release (*5*). In humans, as inhibin B rises and FSH falls, only the most mature follicles continue through the cycle, resulting in a single dominate follicle, and with this selection comes the molecular shift from inhibin B to inhibin A (*6*–*7*). The evolution and absolute imperative of inhibins A and B is to provide mammals with constant negative feedback pressure during the follicular switching that occurs each cycle. If either the signals or the receptors are mutated, reproductive cycles are lost (*1*, *2*).

With two structurally and functionally related but temporally spaced molecules, one may surmise that the pituitary must have some need to integrate the two signals and that turns out to be the case. The inhibin A co-receptor was identified years ago as betaglycan, which sequesters the receptors for the positive signaling pituitary activin and antagonizes its action (*8*), but deletion of this receptor had no impact on FSH release (*9*). The inhibin B co-receptor TGFBR3L is expressed exclusively in the pituitary gonadotroph cell and provides specificity to the B form of inhibin. Both co-receptors curtail activin, whose receptors were identified nearly 20 years ago and assemble a neat package of binding and signal activating serine-threonine receptors (*1*, *4*, *8*, *9*).

Knowing the molecular components of the reproductive cycle, can we speculate on why this cycle is so complex? Brule *et al*. (*1*) speculate that the potential for TGFβ competition created an evolutionary pressure, which then dictated the development of a two-inhibin system of negative feedback. This is clearly in play, but there may be a more pressing evolutionary necessity. Namely, the development of a multistage cycle that places limits on the number of eggs ovulated thereby delimiting the reproductive years. New experiments from the group will likely reveal additional parts of the signaling pathways; but for now, the final piece of a grand negative feedback system has been illuminated with the identification of TGFBR3L as the inhibin B co-receptor, and with it, new possibilities for human reproductive health are on the horizon.

Human reproductive technologies have relied on recombinant FSH as the main tool of stimulating follicles, and, as was described above, overcome the inhibin B break in the system, resulting in a full cohort of follicles and therefore multiple mature eggs. Easy, elegant endocrinology except for the fact that producing large numbers of eggs is not the goal, and IVF remains incredibly inefficient. Instead of quantity, the field of fertility regulation needs quality—a single fertilizable, high-quality egg. The key to the next breakthrough in the field could be directly managing endogenous FSH and its very specific glycosylated variants that are differentially regulated at different cycle stages through inhibins A and B (*10*). Indeed, creating a suite of next-generation small molecules that regulate release and inhibition of endogenous FSH should create more tailored interventions compared to the approaches of today. In this way, inhibin B and its co-receptor close one chapter and usher in a new generation of discoveries.

## References

[1] E. Brule , TGFBR3L is an inhibin B co-receptor that regulates female fertility. Sci. Adv. 7, 51 (2021). 10.1126/sciadv.abl4391 PMC8673766 34910520

[2] Y. Makanji, J. Zhu, R. Mishra, C. Holmquist, W. P. S. Wong, N. B. Schwartz, K. E. Mayo, T. K. Woodruff, Inhibin at 90: From discovery to clinical application, a historical review. Endocr. Rev. 35, 747–794 (2014). 25051334 10.1210/er.2014-1003 PMC4167436

[3] N. Schwartz, C. P. Channing, Evidence for ovarian “inhibin”: Suppression of the secondary rise in serum follicle stimulating hormone levels in proestrous rats by injection of porcine follicular fluid. Proc. Natl. Acad. Sci. U.S.A. 74, 5721–5724 (1977). 271996 10.1073/pnas.74.12.5721 PMC431865

[4] W. Vale, E. Wiater, P. Gray, C. Harrison, L. Bilezikjian, S. Choe, Activins and inhibins and their signaling. Ann. N. Y. Acad. Sci. 1038, 142–147 (2004). 15838109 10.1196/annals.1315.023

[5] H. A. Kenny, T. K. Woodruff, Follicle size class contributes to distinct secretion patterns of inhibin isoforms during the rat estrous cycle. Endocrinology 147, 51–60 (2006). 16195413 10.1210/en.2005-0242

[6] N. A. Klein, B. S. Houmard, K. R. Hansen, T. K. Woodruff, P. M. Sluss, W. J. Bremner, M. R. Soules, Age-related analysis of inhibin A, inhibin B, and activin A relative to the intercycle monotropic follicle-stimulating hormone rise in normal ovulatory women. J. Clin. Endocrinol. Metab. 89, 2977–2981 (2004). 15181087 10.1210/jc.2003-031515

[7] T. K. Woodruff, L. M. Besecke, N. Groome, L. B. Draper, N. B. Schwartz, J. Weiss, Inhibin A and inhibin B are inversely correlated to follicle-stimulating hormone, yet are discordant during the follicular phase of the rat estrous cycle, and inhibin A is expressed in a sexually dimorphic manner. Endocrinology 137, 5463–5467 (1996). 8940372 10.1210/endo.137.12.8940372

[8] Y. Makanji, K. L. Walton, M. C. Wilce, K. L. Chan, D. M. Robertson, C. A. Harrison, Suppression of inhibin A biological activity by alterations in the binding site for betaglycan. J. Biol. Chem. 283, 16743–16751 (2008). 18397882 10.1074/jbc.M801045200

[9] Y. Li, J. Fortin, L. Ongaro, X. Zhou, U. Boehm, A. Schneyer, D. J. Bernard, H. Y. Lin, Betaglycan (TGFBR3) functions as an inhibin A, but not inhibin B, coreceptor in pituitary gonadotrope cells in mice. Endocrinology 159, 4077–4091 (2018). 30364975 10.1210/en.2018-00770 PMC6372943

[10] H. Wang, M. Larson, A. Jablonka-Shariff, C. A. Pearl, W. L. Miller, P. M. Conn, I. Boime, T. R. Kumar, Redirecting intracellular trafficking and the secretion pattern of FSH dramatically enhances ovarian function in mice. Proc. Natl. Acad. Sci. U.S.A. 111, 5735–5740 (2014). 24706813 10.1073/pnas.1321404111 PMC3992661

